# Evaluation of the Display of Cognitive State Feedback to Drive Adaptive Task Sharing

**DOI:** 10.3389/fnins.2017.00144

**Published:** 2017-03-28

**Authors:** Michael C. Dorneich, Břetislav Passinger, Christopher Hamblin, Claudia Keinrath, Jiři Vašek, Stephen D. Whitlow, Martijn Beekhuyzen

**Affiliations:** ^1^Industrial and Manufacturing Systems Engineering, Iowa State UniversityAmes, IA, USA; ^2^Honeywell LaboratoriesGolden Valley, MN, USA; ^3^Human-Centered Systems, Honeywell InternationalBrno, Czechia; ^4^Delft University of TechnologyDelft, Netherlands

**Keywords:** adaptive human-automation systems, neuroergonomics, crew resource management, teamwork, human-computer interaction, cognitive state assessment

## Abstract

This paper presents an adaptive system intended to address workload imbalances between pilots in future flight decks. Team performance can be maximized when task demands are balanced within crew capabilities and resources. Good communication skills enable teams to adapt to changes in workload, and include the balancing of workload between team members This work addresses human factors priorities in the aviation domain with the goal to develop concepts that balance operator workload, support future operator roles and responsibilities, and support new task requirements, while allowing operators to focus on the most safety critical tasks. A traditional closed-loop adaptive system includes the decision logic to turn automated adaptations on and off. This work takes a novel approach of replacing the decision logic, normally performed by the automation, with human decisions. The Crew Workload Manager (CWLM) was developed to objectively display the workload between pilots and recommend task sharing; it is then the pilots who “close the loop” by deciding how to best mitigate unbalanced workload. The workload was manipulated by the Shared Aviation Task Battery (SAT-B), which was developed to provide opportunities for pilots to mitigate imbalances in workload between crew members. Participants were put in situations of high and low workload (i.e., workload was manipulated as opposed to being measured), the workload was then displayed to pilots, and pilots were allowed to decide how to mitigate the situation. An evaluation was performed that utilized the SAT-B to manipulate workload and create workload imbalances. Overall, the CWLM reduced the time spent in unbalanced workload and improved the crew coordination in task sharing while not negatively impacting concurrent task performance. Balancing workload has the potential to improve crew resource management and task performance over time, and reduce errors and fatigue. Paired with a real-time workload measurement system, the CWLM could help teams manage their own task load distribution.

## Introduction

The capacity of the existing Air Traffic Management (ATM) systems are restricted due to current procedures and the workload limitations of air traffic controllers (Quon, 2010). Workload is generally defined as the attentional, cognitive, or response resources required by the human element of a human-machine system to accomplish task requirements (Hart and Wickens, 1990). Yet air traffic demand is expected to more than double between 2015 and 2035 (IATA, 2016). Innovations in the ATM system will be needed to accommodate the expected increase in traffic.

To meet the challenges of future ATM environments, programs like SESAR (SESAR Consortium, 2006) and NextGen (NextGen, 2007) seek to accommodate the air traffic growth and prepare for the demand of 2,020 and beyond. These programs aim to develop new technological capabilities, more automated visualization and decision aids, changes in procedures, and increases in pilot roles and responsibilities. New concepts like precision 4D path following, self-separation, and closer aircraft spacing will be needed to increase capacity and efficiency. Given the expected changes, pilots will be faced with managing increased levels of automation, multiple communication methods, and increased decision making responsibilities. The increased information integration requirements and automation management required by these future systems will increase pilot susceptibility to dangerous deficiencies of situation and automation awareness. Some prominent human-automation interaction problems are likely to increase: uneven distribution of workload, inappropriately aligned trust in automation, breakdown in mode and automation awareness, delays in finding, interpreting and integrating information, and human input errors (Sarter et al., 1997).

Higher functioning teams have a level of mutual organization awareness (Entin and Entin, 2000) that measure the level of awareness each team member has of other's tasks and activities. In team cognition, this is conceptualized as a shared mental model of each other's activities (MacMillan et al., 2004). Team performance will be maximized when task demands are balanced within a team's capabilities and resources (Bowers and Jentsch, 2005). Good information management skills enable teams to adapt to changes in workload, and include the balancing of workload between team members (Hutchins et al., 1999). A definition of team workload has been slow to develop but usually is a combination of individual team member's workload plus the demands needed to coordinate within the team (for a review, see Salas et al., 2008). Effective team performance requires the balance of the task work of individual team members to meet task demands, and the team work needed to coordinate the cooperative efforts of the team (Bowers et al., 1997). This leads to the conclusion that team work adds to the resource demands on the team beyond the demands of the task work (Bowers et al., 1997). However, resource allocation theory would suggest that the resources used to monitor, detect, and address the onset of a workload imbalance are drawn from those resources available to meet the task demands (Porter et al., 2010).

Crew Resource Management (CRM) was developed to improve air safety by focusing on the cognitive and interpersonal skills needed to make optimal use of resources (Helmreich et al., 1999). One of the core function of CRM is to manage the task, resources, and workload of the crew. The goal is to achieve situational awareness and effectively manage the workload distribution of crew members (Kanki, 2010). The management function of CRM is dependent on several factors including the interpersonal atmosphere of the cockpit, crew expectations, available information, and the ability of crewmembers to stay situationally aware (“ahead of the airplane”). A two-pilot crew continually moves between periods of working in parallel, working together, and working alone. Lack of communication can compromise the coordination of crew actions, and lead to periods of mismanagement of crew resources, task timing, and workload distribution (Kanki, 2010). Effective crews have been shown to distribute tasks to avoid overloading individuals (Ruffell Smith, 1979). Markers of observable behavior of interpersonal communication include the clear communication and acknowledgment of the distribution of workload, and the prioritization tasks (Helmreich et al., 1999; Kanki, 2010).

However, CRM typically assigns responsibilities rather than individual tasks, thus relative workloads of the two pilots can often be asymmetric. Likewise, the experience levels of the two pilots may be different. Less experienced pilots may experience higher levels of workload more frequently. Individual tasks are assigned only when one of the pilots becomes overwhelmed or when an abnormal situation occurs. Some airlines have instituted policies to minimize the impact associated with asymmetric workloads. Typically, such policies are not automated and rely on explicit, albeit subjective, criteria to determine when one pilot should offload some tasks to the other.

Although the above-mentioned policies are workable and generally provide desired results, there is room for improvement. There is evidence that some pilots, due to company culture, authority hierarchies, cultural differences, personality, or other factors, may be reluctant to acknowledge that they are overloaded (Helmreich et al., 1999; Engle, 2000). The personality type of the captain can also effect crew performance (Chidester et al., 1990). Crews with captains who had lower motivation of goals and little regard for interpersonal issues initiated communication proportionally less than captains with higher motivation and/or higher regard or interpersonal aspects of crew performance (Kanki et al., 1991). Moreover, pilots may fail to notice that the other pilot has become overloaded, since workload monitoring is a task that itself could be compromised by high workload. Thus, the pilots forego opportunities where the reallocation of tasks could maintain a more optimal workload balance between the pilots.

An operator-initiated adaptive system was developed to objectively determine the workload of multi-pilot crews, notify the pilots, and recommend task sharing or automate lower order tasks, as needed. The Crew Workload Manager (CWLM) concept was designed to help pilots observe the individual and relative workload distribution between two pilots in an effort to improve the capability of flight crews to recognize workload imbalances and subsequently re-allocate tasks during periods of sustained workload imbalance. Balancing workload and reducing the time spent in high workload has the potential to lead to improved crew performance over time, fewer errors, and less fatigued pilots. The relationship between workload and fatigue is complex and the optimal level of workload may change over time (Grech et al., 2009). Both underload and overload can cause fatigue, depending on the circumstances (Hancock and Verwey, 1997). Sustained effort over a long duration produces discomfort and people avoid it whenever possible (Wickens, 1986). Prolonged cognitive workload is seen as a major source of fatigue (Hockey et al., 1989).

The CWLM can display a real-time measure of workload. Previous research has shown that psychophysiological measures can be used to derive accurate estimates of operator cognitive states (Hancock et al., 2013). Cognitive workload assessment can be achieved by many methods. Cardiac, or electrocardiogram (ECG), measures include heart-rate variability (Kalsbeek and Ettema, 1963), tonic heart rate (Wildervanck et al., 1978), variability in the spectral domain (Wilson and Eggemeier, 1991), and T-wave amplitude (Heslegrave and Furedy, 1979). fNIR spectroscopy measures cognition-related hemodynamic changes, and has been used to assess cognitive state (Izzetoglu and Bunce, 2004). Scerbo (1996) concluded that EEG was the most promising of the possible neurophysiological and physiological measures. The success of EEG-based methods has led to an emphasis on the development of more robust EEG measurement devices and classification algorithms (Byrne and Parasuraman, 1996; Prinzell et al., 2003; Wilson and Russell, 2003; Dorneich et al., 2008).

The CWLM acts as an objective, non-threatening third party that displays the assessment of cognitive workload of each pilot. Research has shown that pilots can be unrealistic about the effects of stressors on their performance, and CRM was designed to address these attitude of personal invulnerability (Helmreich and Merritt, 2001). Lack of communication can affect the crew's ability to coordinate tasks (Kanki, 2010). Inappropriate task management and task shedding as a result of breakdowns in crew communications has been shown to be equally prevalent for both novice and experienced pilots (Williams et al., 1993). By acting as an “honest broker,” an assessment of cognitive workload might be better received and responded to than if one of the pilots insinuates that the other pilot is overloaded or unable to handle the current task demands.

The next sections describe the CWLM and Shared Aviation Task Battery (SAT-B). SAT-B was developed as a testbed to study CRM, and was used to manipulate workload between a two-member crew. Finally, an experiment that utilized the SAT-B to evaluate the effects of the CWLM on pilot performance is described and results are discussed.

## The crew workload manager

### The adaptation

The CWLM displays current pilot workload (Dorneich et al., 2011). For the work presented in this paper, cognitive state was manipulated using the SAT-B (see next section). This enabled the experimenters to assess the validity of displaying the workload distribution to pilots via the CWLM without confounding the results with the accuracy of the cognitive state assessment itself (an area of future work). For reference, previous work with EEG and ECG achieved an overall classification accuracy >90% (Dorneich et al., 2007).

**Figure 1:**
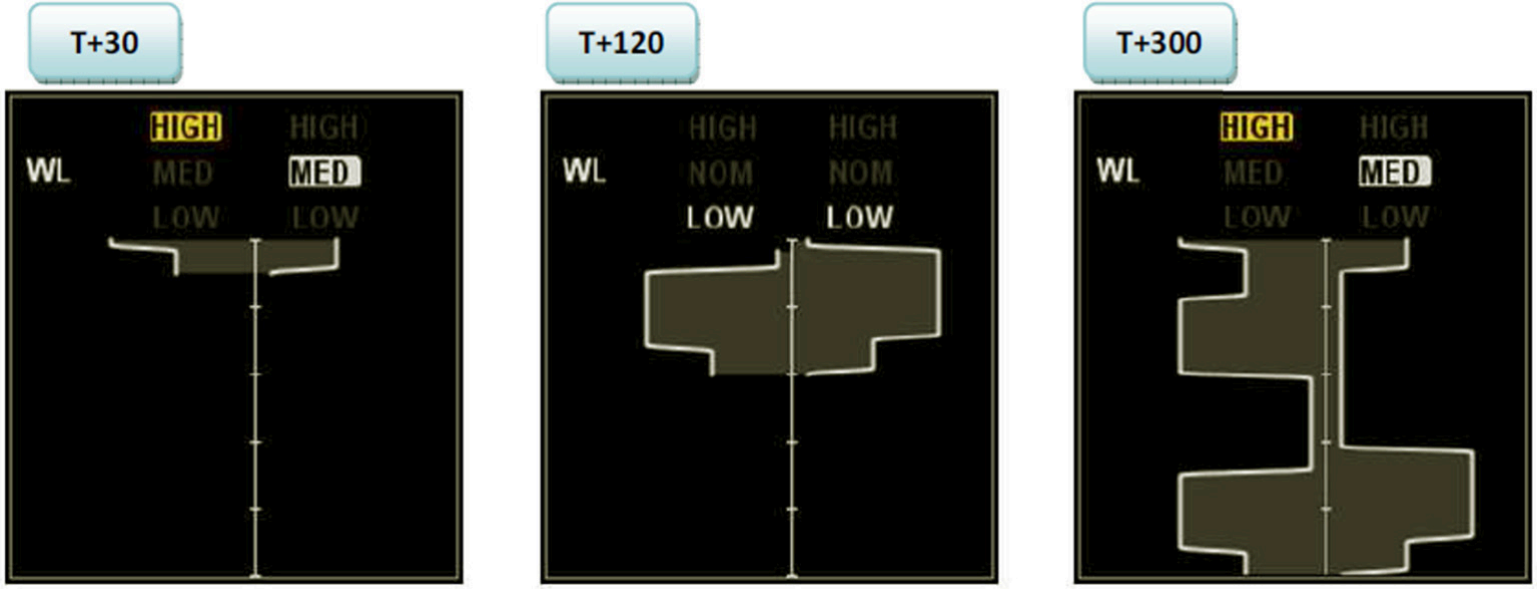
**Crew Workload Manager main user interface at 30, 60, and 300 s after start**.

The CWLM display is illustrated in Figure 1. The CWLM depicts workload for both pilots. Workload for the left operator is depicted left of the display's centerline; workload for the right operator is depicted right of the display's centerline. At the top of the display, the current categorized workload state of each pilot is displayed. The CWLM displays three workload states: low, medium, and high. High workload was operationalized as workload at or near the maximum resource capacity of the operator, where they would not be able to take on an additional task without a decrease in overall performance. Thus a pilot could be at high workload but still be performing well. Conversely, low workload can be defined as times when the participant has the resources to easily take on additional tasks (Dorneich et al., 2008).

A 5-min history of workload is displayed as a timeline running from top (newest) to bottom (oldest). Low workload is indicated by a narrow band closest to the centerline while high workload is indicated by wide band furthest from the centerline.

**Figure 2:**
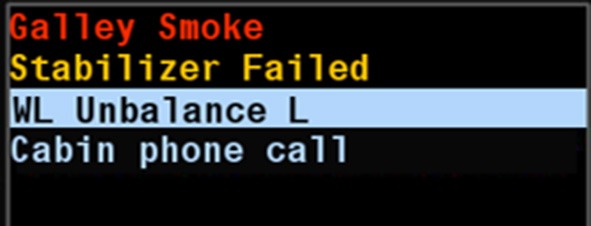
**Example alert message associated with the CWLM**.

When workload is out of balance between operators, or if workload for one of the operators was determined as “High” an advisory notification triggered an alert message in the crew alerting system (CAS) window (see Figure 2). In the case of a workload imbalance, the CAS window displayed the text “Workload imbalance L (or R).” “L,” and “R” indicated which pilot was experiencing high workload. The CAS messages were triggered only in case of a High-Low or Low-High workload distribution, where the situation may have been solvable by task sharing. Medium-High and Medium-Low combinations were not considered situations where the CWLM would actively intervene as task sharing may be costly or inappropriate.

### CWLM

A traditional closed-loop adaptive system includes three principle elements (Feigh et al., 2012): (1) measurement of workload in real time to act as triggers for adaptations, (2) decision logic to decide when to turn on and off automated adaptations based on the triggers, and (3) the adaptations themselves in of form of changes to the automation and human-machine interface. This work takes a novel approach of replacing the decision logic, normally performed by the automation, with human decision logic. In this scenario, a measurement of workload would be displayed to the pilots, who then “close the loop” themselves by deciding how to best mitigate an unbalanced workload between pilots. With the CWLM, it is up to pilots to address the situation by adapting their workload distribution. The automation is not the initiating agent of changes to the task environment. The CWLM simply displays the workload imbalance and recommend task redistribution, and it is up to the human operator to initiate any changes to mitigate the condition of concern.

## The shared aviation task battery

The SAT-B was developed for this evaluation as a testbed to study CRM. The SAT-B was inspired by the well-established experimental Multiple Attribute Task Battery (MAT-B) test bed, which was designed to evaluate single operator performance and workload via a set of aviation-related tasks (Comstock and Arnegard, 1992). In contrast, the SAT-B was designed to allow two people to each have screens with identical content, where tasks were shared between the two operators, similar to the redundant displays in two-pilot cockpits (e.g., primary flight display). The control of each task is assigned individually to a participant. Participants are taught that if they feel their performance on a task is deteriorating, they may off-load a task to the other participant. Likewise, if a participant feels his or her partner is overwhelmed or performance is deteriorating, the participant can also help his or her partner by taking over a task. In this way the two participants share tasks and dynamically decide how to distribute the tasks between themselves. Thus the SAT-B can be used to study the joint performance, coordination, and resource management between two operators. The SAT-B simulates five simple cognitive tasks running in parallel, much like MAT-B. Task load is manipulated by changing the rate at which events happen or rate and magnitude of deviation forces. The five tasks are:

- *Monitoring Lights (ML)*. The participant monitors two indicators (green and red). When the green light goes off the participant has to turn it back on again. When the red light turns on, the participant turns it off.
- *Tracking (T)*. Participants must continually compensate for course deviations of the aircraft by keeping a target symbol inside a prescribed rectangular box in the both the x- and y-direction, while semi-random disturbances force the aircraft from the straight and level condition.
- *Monitoring Dials (MD)*. The participant monitors four analog gauges representing manual engine thrust control. When random system malfunctions cause the values to deviate, the participant corrects them to keep the values in the appropriate range.
- *Resource Management (RM)*. The participant monitors and controls the fuel levels in two tanks pairs within a given range via a system of tanks and pumps, each with different flow rates.
- *Communications (C)*. The participant monitors air traffic radio chatter and responds only to messages preceded by their call sign, and tune the radio frequency or navigation aid frequency per ATC's instruction.

### Interface

**Figure 3:**
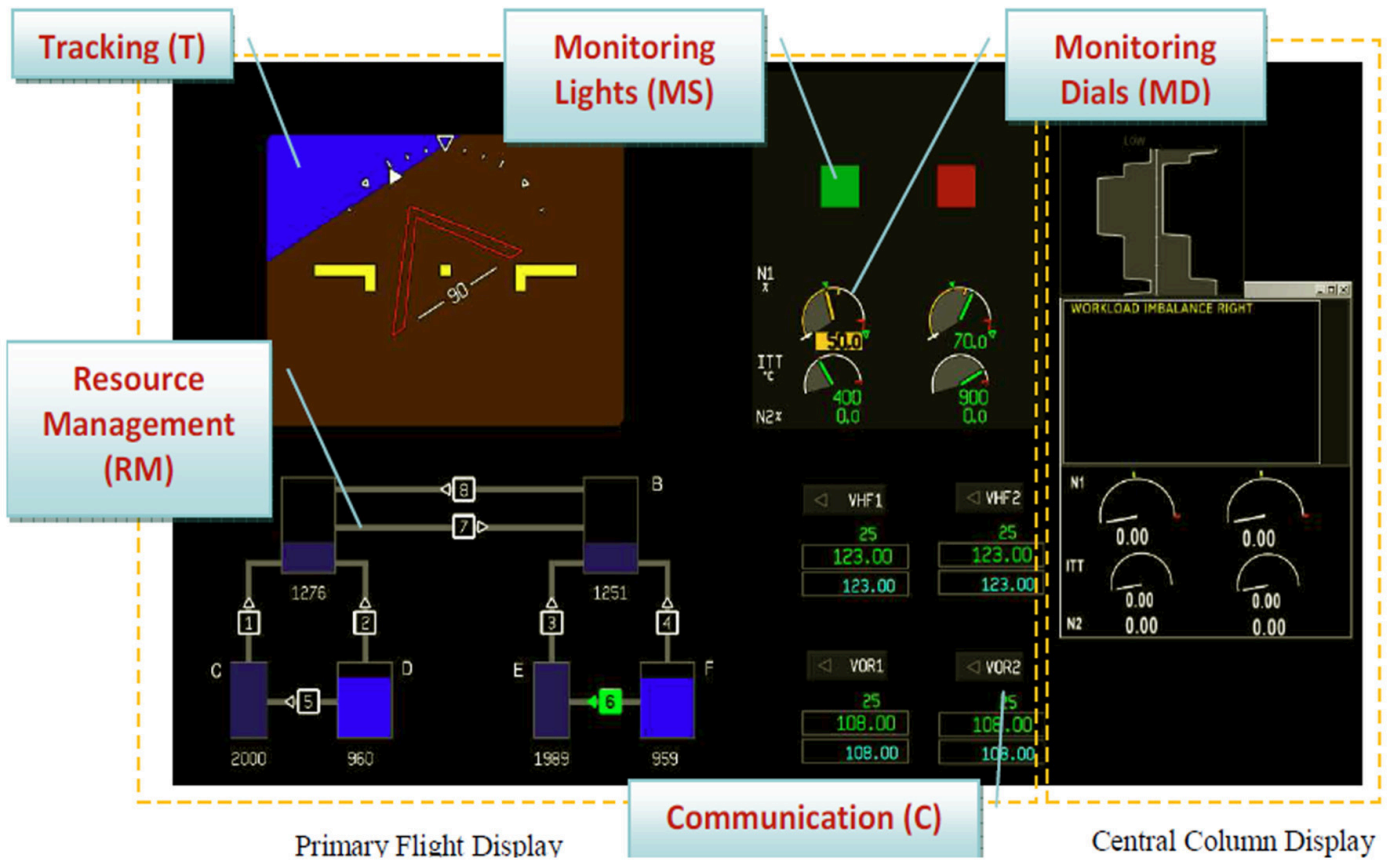
**The SAT-B is designed for dual operation between two participants**.

The SAT-B interface is shown in Figure 3. The tracking task is shown in the upper left hand corner. The dial indicators used to perform the monitoring task are in the upper right hand corner of the display. The resource management task is shown in the lower left area of the display. The communications task is shown in the lower right hand area of the display.

### Pilot study: manipulation of workload

In addition to SAT-B providing a platform to study task sharing, it can also be used to manipulate participants' workload. By varying the event rate of the five tasks, participants can be put into a state of low, medium, and high workload. Pilot tests were conducted to determine the appropriate task rates. The goal was to find rates for each task that resulted in different levels of workload but were not so hard that the participants would give up trying to perform the task. Thus the highest workload chosen was designed to be below the threshold at which performance would degrade. Each of five different SAT-B tasks was tested at three rate levels. These task/rates combinations where then grouped together to create groups of tasks at particular rates. For instance, combining Monitoring Lights and Tracking tasks, each at a low rate, results in a low combined workload; but combining Monitoring Lights and Tracking at low rates plus Communication at a high rate results in overall medium workload. Three participants rated each group of tasks using a NASA TLX scale. Groups were then chosen to form low, medium, and high task/rate combinations for use in the study. Some groups were considered borderline between two workload levels and were not used.

The pilot study determined the distribution of tasks (and each task rate) between two users to produce levels of low, medium, and high task load. The communication task was chosen as the task that could be exchanged between the two participants because there was little or no spin-up costs to taking over the task. Of the remaining four tasks, it was determined that Monitoring Lights and Tracking tasks would be paired for one participant, while the other participant conducted Monitoring Dials and Resource Management tasks. Thus each paring contained one continuous and one discreet monitoring task to keep the attention demands of the two task distributions as similar as possible. Finally, the pilot study determined that 30 min of practice time enabled participants to become practiced in the SAT-B tasks, with negligible learning effect with subsequent practice. This was used to set the training and practice time in the experiment at 60 min to ensure there was no learning effect.

## Material and methods

An evaluation was performed to assess whether the CWLM would improve CRM.

### Objective and hypotheses

It was hypothesized the CWLM would enable the participant to better recognize imbalanced workload conditions and to respond faster by either on-loading or off-loading tasks to their colleague (a confederate), resulting in a more balanced workload between the operators. While the CWLM was not expected to improve task performance, it is important to make sure that task performance is not decreased as a result of the increased emphasis on task sharing. The experiment was conducted in order to evaluate three hypotheses related to the potential benefits and costs of the approach:

- The CWLM adaptation will decrease the amount of time in unbalanced workload (benefit).
- The CWLM adaptation will increase the appropriateness of task sharing requests between two crew members. (benefit).
- The addition of the CWLM adaptation will not negatively affect crew performance on concurrent tasks. (cost).

In addition, participants were asked a series of questions to understand their opinion of the CWLM.

### Participants

Six male participants took part in the experiment. The six participants ranged in age from 30 to 37 years (*M* = 32.5, *SD* = 2.9). One participant held a private pilot license, three had experience riding jump seat on airliners, and five participants were familiar with glass cockpit avionics through flight simulators. All participants were trained to use SAT-B. This study was carried out in accordance with the federal regulations of the Czech Republic with approval from the EU ARTEMIS JU commission for all subjects. All subjects gave written informed consent in accordance with the Declaration of Helsinki.

The SAT-B was conducted by a “crew” of two: an experiment participant and a confederate. A confederate is an actor who is part of the experimental team and knows the aims of the study. A confederate was used in order to exert more control on the task load manipulation of the participant. Participants were not aware that the second operator was a confederate.

### Equipment

The SAT-B software was installed in a fixed-based flight simulator of an A320 airplane. There were two pilot seats, where the SAT-B monitor was in the primary field of view and the CWLM display was located on the upper central pedestal display unit. The CWLM workload values were driven by the SAT-B task loads. The SAT-B software was used to manipulate the participant workload, based on pilot studies that established the event rates necessary to induce low, medium, or high workload.

### Tasks

Each scenario started with an initial assignment of the five SAT-B tasks between the participant and the confederate. As the scenario progressed, the tasks varied in their cognitive load (due to manipulations of their event rates), with the concomitant change in participant workload. It was then up to the participant to on- or off-load tasks depending on his or her assessment of his or her own workload and the workload of their partner. Participants were required to recognize when they were overloaded and pass off tasks to the confederate if that pilot had spare capacity. Conversely, the participant also had to recognize when the confederate was overloaded and actively take on tasks.

Participants were seated in the left seat of a flight simulator for all experiment conditions. Participants were told that the confederate was acting as their partner and that success of the flight was evaluated based on performance of the crew as a whole. Sharing the Communication task was the only means to change the workload distribution between the crew. Either partner could request and/or accept workload sharing queries. Ownership of the shared task was indicated by a green dot presented on the owner's screen.

### Independent variables

There were two independent variables: Initial Task Distribution (A & B) and CWLM Adaptation (On & Off).

#### Task distribution

The two conditions are distinguished by the initial task distribution between the participants and the confederate (Table 1). In Task Distribution A, the participant begins the trial assigned to the Monitoring Lights and Tracking tasks; the confederate has the Monitoring Dials, Resource Management, and Communications tasks. In Task Distribution B, the task assignment is reversed between the participant and the confederate. The Communication task, which is the task designated for sharing, is in the beginning of the experiment assigned together with Monitoring Dials and Resource Management tasks (i.e., Task Distribution B).

**Table 1:** **The task distribution independent variable description**.

**Task**	**Task distribution A**	**Task distribution B**
Monitoring Lights	Participant	Confederate
Tracking	Participant	Confederate
Monitoring Dials	Confederate	Participant
Resource Management	Confederate	Participant
Communications	Confederate (Initially)	Participant (Initially)

#### CWLM adaptation

When the CWLM is off, the participant was expected to determine their own and the confederate's workload through observation of task performance. In the second condition, the CWLM is on and can be used by the participant to assess workload.

### Experimental design

Given the number of participants, the experiment was designed as a 2 (Task Distribution: A, B) × 2 (Adaptation: On, Off) within-subject design. In order to test both the Adaption Off and On conditions with the same subject, the presentation order was fixed, where the Adaptation Off condition was presented first, and the Adaptation ON condition (CWLM) was presented second. To ensure that there was minimal learning effect because of trial order, the participants were given extensive training (60 min, or twice the level found were needed in pilot experiments).

### Experimental trial scenarios

The experimental trials were designed to induce periods of unbalanced workload between the participant and the confederate. The workload of each individual was manipulated by varying the rates of the individual SAT-B tasks. Pilot studies determined that it was possible to reliably induce three distinct workload levels (Low, Medium, High) with various combinations of tasks and their respective event rates.

Table 2 described the experimental trial design. Each column represents a 60 s time block. The second and third rows describe the induced cumulative workload level of the participant and confederate. The remaining rows describe which task was conducted by whom. The number in the cells is the task rate of that task, on a scale of 1 (lower) to 3 (higher). Thus in the column “0” (time block) the participant has a low overall induced workload (“L” in row 2) because he or she is conducting three tasks (M+T+C), each at a lower rate (“1”). Likewise, for this block, the confederate is under medium induced cumulative workload (“M” in row 3) since he or she is conducting two tasks (MD+RM) at a higher rate (“3”). Finally, the gray bocks are the data collection periods where there is a workload imbalance that the participant needs to detect and address.

**Table 2:**
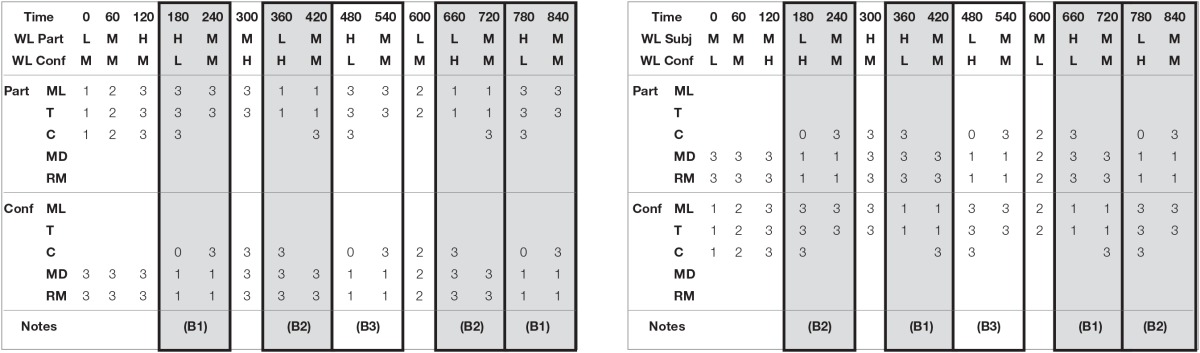
**Trial scenario descriptions for Task Distribution A (left) and Task Distribution B (right)**.

*The gray areas mark the data collection periods and are situations where the participant needed to detect and mitigate unbalanced workload. The individual task rate (1–3) of each task is described in the cells*.

Trial scenarios were designed to smoothly change the workload of an individual by only changing the task load by a maximum of one rate level for one task at any one time, to prevent a discernible “jump” that would serve to alert the individual that the task load had changed. Thus the trial moved through a series of task load changes over time. Each of four trials lasted 15 min, and the task load was manipulated by a computer script which changed task rates in a predefined manner. Each script (or scenario) made changes in the combined task load every 60 s.

Within each scenario there were five 2-min blocks where the workload became unbalanced. Participants were required to detect the imbalance and reallocate tasks. If the participant did not detect the imbalance within 60 s; the confederate was instructed to intervene by either offering to take a task or asking to share a task. In one of the five unbalanced blocks, the confederate would offer or ask for assistance immediately at the beginning of the unbalanced block in order to the keep the illusion that he operated under the same rules as the participant. The time limit of 60 s was enough time for a participant to detect that he or she was under high workload, or to notice the confederate under high workload, while still allowing multiple data collection opportunities. Thus each 15-min scenario provided four opportunities to collect data on how long it took the participant to detect and fix an imbalance of workload. The exact distribution of five unbalanced blocks is given also in Table 2, where unbalanced block types are marked as follows:

- (B1) Unbalanced—Confederate (C) offers help after 60 s if Participant (P) does not ask before then
- (B2) Unbalanced—Confederate (C) asks for help after 60 s if Participant (P) does not offer before then
- (B3) Unbalanced—Confederate (C) offers/asks for help immediately at beginning of unbalanced block (data not included in calculations).

Sharing requests by the participants from blocks B1 and B2 can be either correct or incorrect (depending on the direction of the request). The block B3 was included to provide the confederate a chance to request a change the task distribution, so the participant would not get suspicious that the confederate never took the initiative. Data from the B3 block was therefore not included in the calculations of results. Sharing requests by the participant from any block not labeled B1, B2, or B3 were incorrect, and were rejected by the confederate. It should be noted that the direction of workload distribution when making a request (participant is in low workload vs. participate is in high workload) may be a “hidden” independent variable in the evaluation. However, all results were tested against this possibility, and the direction of workload distribution was not significant for any results, and thus it was not considered an independent variable in the results.

### Dependent variables

Dependent variables will be: (1) time spent in unbalanced workload, (2) Number of correct sharing requests, (3) number of incorrect sharing requests, (4) measures of performance on the five SAT-B tasks, and (5) ranking between the workload of the trials.

Total time spent in an unbalanced workload state was considered the most indicative of the impact of CWLM on CRM. The measure was defined as sum of time spent in unbalanced workload during the trial.

The correct requests count was defined as the number of times the participant correctly asked to change the task distribution (both asking to offload a task and offering to accept a task). The related measure incorrect requests count was defined as number of requests to change the task distribution (both asking to offload a task and offering to accept a task) in situations when such activity would be unnecessary and therefore a distraction. As such, the incorrect request count was expected to be related to the potential negative impact of CWLM on workload, performance, and a potential indicator of insufficient training in the sharing procedures. The experimental scenario design contained three different blocks of unbalanced workload (B1, B2, B3). Sharing request are correct or incorrect as summarized in Table 3.

**Table 3:** **Conditions for a correct or incorrect sharing request**.

**Participant**	**B1**	**B2**	**B3**	**Other Blocks**
Asking to offload a task	Correct	Incorrect	(data not used)	Incorrect
Offering to accept a task	Incorrect	Correct	(data not used)	Incorrect
Does not ask or offer	Incorrect	Incorrect	(data not used)	Correct

The measures of performance on the five SAT-B tasks were as follows:

- Median reaction time for Monitoring Lights (red, green) and Monitoring Dials tasks (red, green)
- Mean processing time for Communications task.
- Number of errors for Monitoring Lights (miss, FA), Monitoring Dials (miss, FA, incorrect entries), and Communications (miss, FA, incorrect entries) tasks.
- Deviations for Tracking (integral of deviation from center) and Resource Management (integral of deviation out of dead band zones).

Pilot testing established the SAT-B task rates needed to manipulate the task load of participants throughout the trial, which were changing every 60 s. It was impractical to interrupt participants every 60 s during each trial to take measures of subjective workload. In order to establish if the subjects felt differences in overall workload of each trial, participants were asked at the end of the experiment to rank in order the overall workload of each trial relative to each other. In other words, participants assigned a rank of 1 through 4 to the four trials, where the rank of “1” was assigned to the trial with the highest workload, the rank of “2” was the second highest workload trial, and so on. The predicted order of the workload trials (from highest workload to lowest) was Trial 2 > Trial 1 > Trial 4 > Trial 3.

### Data analysis

The data was tested for the normality assumption using the Shapiro-Wilk test. Data found to be normally distributed was analyzed using Analysis of Variance (ANOVA) tests to test for statistical significance. Data not found to be normally distributed was analyzed using Wilcoxon rank scores. If the factor has two or more levels, the Kruskal-Wallis test is performed. Results are reported as significant for alpha <0.05. Cohen's d is an effect size that indicates the standardized difference between mean of two groups (Cohen, 1988). Cohen's d results are reported as small for 0.20 < d < 0.50, medium for 0.50 < d < 0.80, and large for d > 0.80. Page's Trend Test was used to test if the ranking of the trial workload was significantly correlated between participants. It is a repeated measures comparison of ordered correlated variables and is useful when there are three or more conditions, the judges (participants) see every condition, and there is a predicted order of the ranking (Page, 1963).

### Protocol

The study was performed with each of the participants individually. Participants were briefed on the CWLM concept, the importance of balancing workload, and trained on the SAT-B tasks. After 60-min training session for each of the two different task combinations (A, B) participants conducted an hour of training on how to share tasks. The four experimental trials each lasted 15 min, with a 5-min break in between each. After the trials were completed, the participant filled out a survey to give subjective feedback on the CWLM.

## Results

### Unbalanced workload

**Figure 4:**
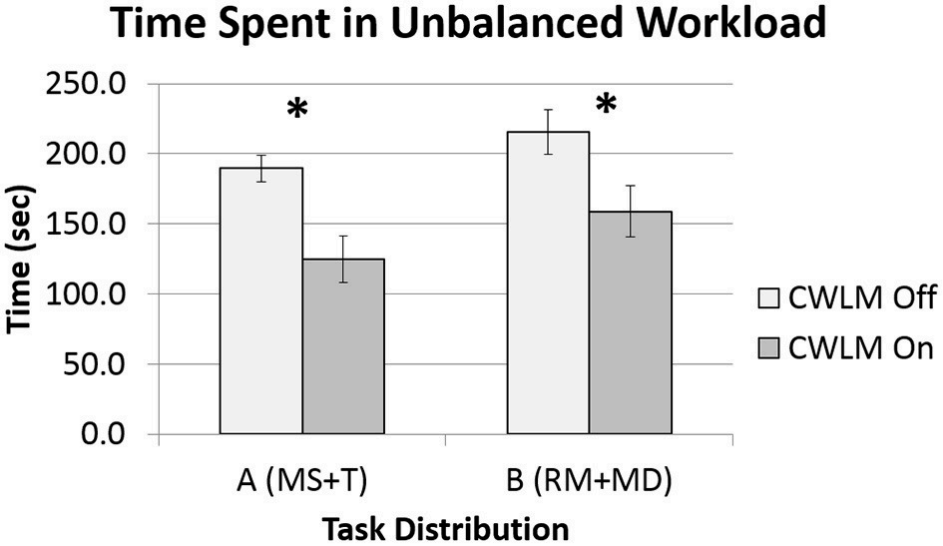
**Means and standard error bars for time spent in unbalanced workload**. The star “^*^” indicates a significant difference between CWLM adaptation levels.

One of the four data sets was found to not be normal, and so Wilcoxon tests were performed. There was no significant (*Z* = 1.64, *p* = 0.10) difference between task distributions A and B. The time spent in unbalanced workload for CWLM-On trials was significantly (*Z* = 2.92, *p* = 0.004, d = −1.5) less than the time in CWLM-Off trials. For Task Distribution A, the time dropped from 189.7 (*SD* = 23.3) seconds with CWLM Off to 124.7 (*SD* = 40.2) seconds with CWLM On; for Task Distribution B the time dropped from 212.2 (*SD* = 37.1) seconds to 161.2 (*SD* = 42.0) seconds (see Figure 4).

### Task sharing requests

**Figure 5:**
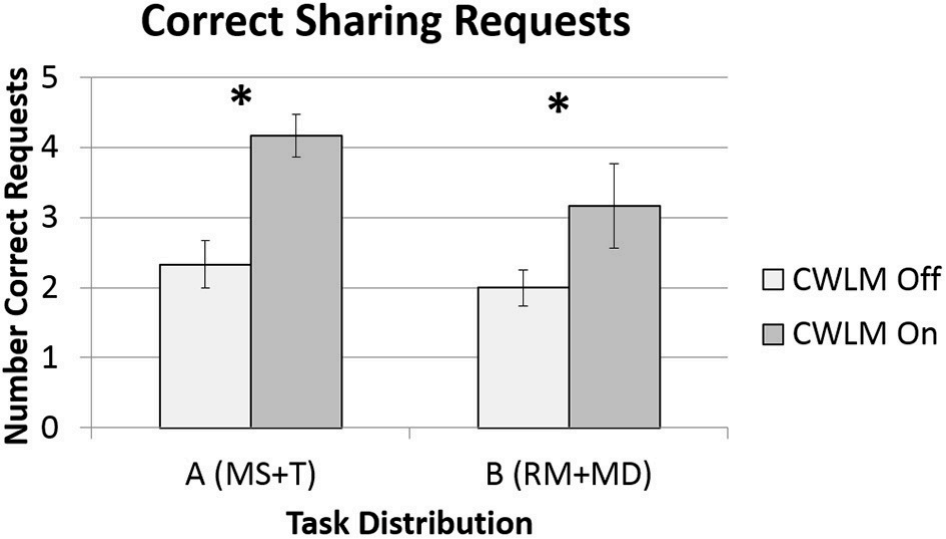
**Means and standard error bars for correct requests for task sharing**. The star “^*^” indicates a significant difference between CWLM adaptation levels.

The data for correct sharing requests was found to be normally distributed, and so an ANOVA was conducted. Figure 5 illustrates the data for all four conditions. There was no significant [*F*_(1, 5)_ = 5.71, *p* = 0.062] difference between Task Distribution A and B. Participants in the CWLM-On condition made significantly [*F*_(1, 5)_ = 19.3, *p* = 0.007, d = 1.9] more correct sharing requests (*M* = 3.67, *SD* = 1.0) than participants in the CWLM Off (*M* = 2.17, *SD* = 0.41) condition.

**Figure 6:**
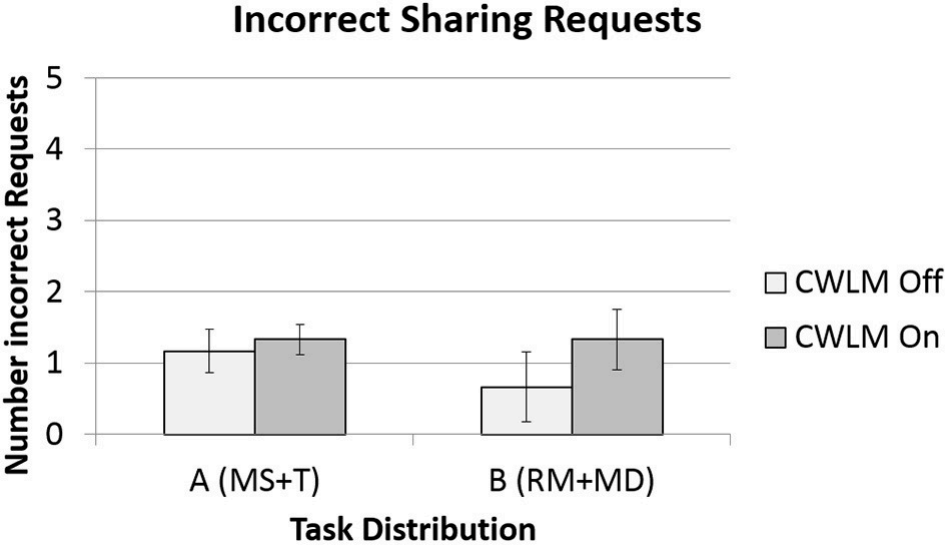
**Means and standard error bars for incorrect requests for task sharing**.

Two of the four data set for in incorrect sharing requests sets was found not be normal, and so Wilcoxon tests were performed. Figure 6 illustrates the data for all four conditions. There was no significant (*Z* = −1.05 *p* = 0.30) difference between Task Distribution A and B. Participants in the CWLM On condition also made more incorrect sharing requests (*M* = 1.33, *SD* = 0.78) than participants in the CWLM Off (*M* = 0.92, *SD* = 1.0) condition, but the difference was not significant (*Z* = −1.26, *p* = 0.21).

### SAT-B tasks

Most of the performance-related data was found to be normally distributed, except for number of errors of monitoring red light, number of errors in communication, reaction time for monitoring dials, and deviation during resource management. None of the SAT-B tasks showed any significant difference in performance of the participant under any of the independent variables. Table 4 illustrates the means for the CWLM Off and CWLM On trials for all the performance metrics associated to the SAT-B tasks, and includes the critical statistic and *p*-value. Results of each task are the participant's performance only, except for the shared task of communications, where the results are for the combined performance of the participants and the confederate.

**Table 4:** **Performance metrics for SAT-B tasks**.

**Task**	**Metric**	**Unit**	**CWLM Off Mean (Std Dev)**	**CWLM On Mean (Std Dev)**	**Critical Statistic**	***p*-value**	**Sig?**
Monitoring lights (ML)—Red	Median reaction time	Sec	1.45 (0.06)	1.46 (0.07)	*F*_(1, 5)_ = 0.36	0.57	No
Monitoring lights (ML)—Green	Median reaction time	Sec	1.48 (0.05)	1.49 (0.07)	*F*_(1, 5)_ = 1.79	0.24	No
Monitoring lights (ML)—Red	Number of errors (miss, FA)	Number	3.25 (1.7)	2.5 (1.4)	*Z* = −0.86	0.39	No
Monitoring lights (ML)—Green	Number of errors (miss, FA)	Number	5.08 (4.0)	6.58 (3.5)	*F*_(1, 5)_= 0.52	0.50	No
Tracking task (T)	RMS of distance deviation from center	Distance	82.5 (26.7)	87.7 (31.4)	*F*_(1, 5)_ = 1.81	0.23	No
Communication (C)	Median command processing time	Sec	1.79 (.34)	1.93 (.52)	*F*_(1, 5)_ = 2.14	0.20	No
Communication (C)	Number of errors (miss, incorrect entries)	Number	1.63 (1.34)	1.17 (1.13)	*Z* = −1.22	0.22	No
Monitoring Dials (MD)	Median reaction time	Sec	11.1 (2.1)	10.8 (1.1)	*Z* = 0.24	0.81	No
Monitoring Dials (MD)	Number of errors (miss only)—97% of all errors	Number	18.6 (4.9)	18.2 (8.3)	*F*_(1, 5)_ = 0.03	0.87	No
Resource Management (RM)	Integral of deviation out of dead band zones	Distance	280 (325)	191 (203)	*Z* = 0.31	0.76	No

### Workload ranking of trials

Participants average subjective rank was Trial 2 (*M* = 3.2, *SD* = .90) > Trial 1 (*M* = 3.0, *SD* = 1.0) > Trial 4 (*M* = 2.0, *SD* = 1.0) > Trial 3 (*M* = 1.8, *SD* = 0.90). The participants average trial order ranking was significant (*L* = 165, *p* < 0.05). Thus the two trials (Trials 2 and 1) without the CWLM were ranked as higher in overall workload than the two with CWLM (Trials 3 and 4).

In follow up interviews, participants reported that the workload displayed matched their own perception of their workload. On the rare occasions when they noticed a discrepancy between the workload displayed by CWLM and their own self-evaluated workload, the CWLM indicated their workload as high, but participant's self-evaluation was medium. Furthermore, participants indicated that they trusted the CWLM assessment.

## Discussion and conclusions

The first hypothesis held that the CWLM adaptation would result in a better overall balance of task load across crew members. This hypothesis was fully supported. Results indicated there was a significant decrease in the amount of time crew members spent in unbalanced workload state when the CWLM was present. The presence of the CWLM allowed participants to recognize more quickly when the task load was distributed unequally, and more quickly initiate sharing activity. As a result, participants were more active in managing crew resources by offering help and asking for help. Without the CELM, participants were prioritizing individual task demands, and spending less attentional resources on the resource management function of CRM. The CWLM offer a type of supporting behavior enabling team members to compensate for each other's weaknesses by shifting workload (Smith-Jentsch et al., 1998). Since workload in another person is often difficult to observe, the opportunity to provide backup for an overload teammate may not arise if that teammate does not communicate his or her need (Smith-Jentsch et al., 1998).

The second hypothesis stated that the CWLM adaptation would increase the appropriateness of task sharing between two crew members. This hypothesis was partially supported. The number of correct sharing requests was significantly higher in the CWLM conditions, and there was no change in the number of incorrect sharing requests. However, the number of incorrect sharing requests was also significantly higher. When comparing the magnitudes of the increases, as well as the effect sizes, the increase of correct sharing requests was 3.6 times greater in magnitude than the increase in incorrect sharing requests. So a large increase in correct sharing requests comes at the cost of a smaller increase in incorrect requests. All the teams in the study were novice teams. However, higher performing teams often have less need of supporting behavior, and would require less sharing requests (Smith-Jentsch et al., 1998).

Finally, the third hypothesis stated that the addition on the CWLM adaptation will not negatively affect crew performance on concurrent tasks. This hypothesis was fully supported. There was no evidence that the addition of a task to monitor the CWLM caused any decrement in any of the task performance metrics across the five STA-B tasks. This is important because both monitoring of crew resource imbalance and workload sharing (potentially new tasks) should not come at the expense of decreased performance of current tasks. The CWLM is not necessarily designed to improve performance immediately. It is hypothesized that prolonged workload imbalance would eventually decrease task performance, and future work is needed test this premise.

In follow-up interviews, all participants indicated that they felt the CWLM helped reduce the difficulty and workload of assessing the other crew member's workload. They felt that the CWLM was easy to comprehend, encouraged its usage, and reduced participant stress related to being assessed by other crew member. A typical participant response was, “I felt I could share [tasks] without uncertainty that I may disturb or cause some trouble.” The CWLM may act as a cognitive prosthesis or tool (Hollan et al., 2000) that offloads some of the teamwork demands. More specifically, the CWLM will monitor, detect, alert, and suggest a mitigation to help crews keep workload in balance, thus relieving them of some of teamwork demands that take up cognitive resources that could be used to meet task demands.

Participants reported the CWLM reflected their actual workload, save for a few rare occasions where it rated medium workload as high workload. Furthermore, their workload rankings significantly correlated to the intended manipulation through SAT-B task rates, indicating that the SAT-B was able to successfully manipulate workload. Confident that the CWLM reflected the participant's true workload (even though it was not being measured directly), the quantitative and qualitative results can be used to assess the efficacy of the CWLM display approach. These results suggest that the presence of CWLM may have been perceived as a validation of the participant's self-assessment of his own workload, as well as an indication of the other person's workload assessment. More, research is needed to understand what accuracy level of real-time workload assessment will be necessary for humans to maintain trust in the CWLM system.

This willingness to accept the CWLM could be taken as indication of the potential acceptability the CWLM to act as an “honest broker” that could overcome human biases to take on more workload than necessary. This has the potential to change the dynamic on the flight deck with repent to CRM. By relying on an automated announcement of workload distribution, the management function of CRM may be less reliant on interpersonal factors that may hinder good communication (Kanki, 2010), as well as keeping everyone situationally aware of each other's workload. however, more research will be needed to assess the acceptability of the CWLM with different types of team operating under different team dynamics.

Overall, participants felt that the CWLM helped them to quickly orient themselves to the other person's workload. However, qualitative feedback made it clear that participants did not use the CAS display. One participant suggested that the CAS could be made more salient, but generally, the CAS messages were not perceived as necessary since all of required information was already present in graphical form in the main CWLM HMI, and was in an easy and quickly understandable.

Beyond the cockpit crew, many domains are interested in maintaining a balance of workload within the team. For instance, air traffic controllers must monitor within own sectors as well as coordinate with other controllers as aircraft transition sectors. Critical situations can quickly create workload imbalances, and there is a need for strategies to balance the workload between team members to manageable levels (Malakis and Kontogiannis, 2008). Balancing workload is an explicit goal in the development of artificial cognition to enhance cooperation of humans unmanned air vehicles (Meitinger and Schulte, 2009).

Future work is also needed to support the premise that long term workload balancing improvements would result in a reduction in fatigue and potential benefits in crew responsiveness to non-normal and off-nominal events. As cognitive state assessment improves in diagnostic accuracy in ever more realistic operational environments, there is the potential to create closed-loop adaptive automation to respond to unbalanced workload (Dorneich et al., 2007). However, such automated interventions need to be designed with an understanding of the interplay between potential near-term benefits of the adaptations and the long term costs that may be associated with use of such systems (Dorneich et al., 2016). For instance, automation could be more directive and recommend or even execute a task reallocation between pilots; however, there is the danger that that the system will lead pilots “down a garden path” and inhibit the critical review of the situation to decide the appropriate response. Automated responses may foster an overreliance on the system's assessment of the situation, and erode pilot skills over the long term. The adaptive nature of the design may address some of these concerns, but more work needs to be done to determine the frequency and level of automated support that balances short term joint performance improvements and long-term performance costs. Additionally, more work needs to be done on the triggering side of the system—the automated interventions are only effective when they are used in the appropriate situations. For any system that uses real-time assessment of cognitive state, there are issues of accuracy, deployability, and user acceptance that need to be addressed before any system like CWLM can be successfully integrated into operational practice.

## Author contributions

MD lead the writing of the manuscript; co-designer of the adaptive system, SAT-B, and evaluation; and did final data analysis. BP lead the pilot studies to establish the SAT-B; co-designer of the adaptive system, SAT-B and evaluation; lead the running of the evaluation; did initial data analysis; and contributed writing to manuscript. CH co-designer of the adaptive system, SAT-B, and evaluation; and contributed writing to manuscript. CK was PI of project; co-designer of the adaptive system, SAT-B, and evaluation; and contributed writing to manuscript. JV was co-designer of the adaptive system and evaluation; helped run the study; and contributed writing to manuscript. SW was co-designer of the adaptive system, SAT-B, and evaluation; and contributed writing to manuscript. MB designed and implemented the interface of the adaptive system.

### Conflict of interest statement

The authors declare that the research was conducted in the absence of any commercial or financial relationships that could be construed as a potential conflict of interest.

## References

[B1] Bowers C. A. Jentsch F. (2005). Team workload, in Handbook of Human Factors and Ergonomic Methods, eds Stanton N. Hedge A. Brookhuis K. Salas E. Hendrick H. (Boca Raton, FL: CRC Press), 57–1–57–3.

[B2] Bowers C. Braun C. Morgan B. (1997). Team workload in Its Meaning and Measurement, in Team Performance Assessment and Measurement: Theory, Methods, and Applications, eds Brannick M. T. Salas E. Prince C. (Mahwah, NJ: Lawrence Erlbaum), 85–108.

[B3] Byrne E. A. Parasuraman R. (1996). Psychophysiology and adaptive automation. Biol. Psychol. 42, 249–268. 10.1016/0301-0511(95)05161-9 8652747

[B4] Chidester T. R. Kanki B. G. Foushee H. C. Dickinson C. L. Bowles S. V. (1990). Personality Factors Inflight Operations, Vol. 1, Leader Characteristics and Crew Performance in Full-mission Air Transport Simulation. Moffett Field, CA: NASA-Ames Research Center. Nasa Technical Memorandum 102259.

[B5] Cohen J. (1988). Statistical Power Analysis for the Behavioral Sciences, 2nd Edn. Hillsdale, NJ: Erlbaum.

[B6] Comstock J. R. Arnegard R. J. (1992). The Multi-attribute Task Battery for Human Operator Workload and Strategic Behavior Research. Hampton, VA: NASA Langley ResearchCenter. Technical Memorandum No. 104174.

[B7] Dorneich M. C. Mathan S. Ververs P. M. Whitlow S. D. (2008). Cognitive state estimation in mobile environments, in Augmented Cognition: A Practitioner's Guide, eds Schmorrow D. Stanney K. (Santa Monica, CA: HFES), 75–111.

[B8] Dorneich M. C. Passinger B. Beekhuyzen M. Hamblin C. Keinrath C. Whitlow S. (2011). The crew workload manager: an open-loop adaptive system design for next generation flight decks, in Proceedings of the Human Factors and Ergonomics Society Annual Meeting (Las Vegas, NV), 19–23.

[B9] Dorneich M. C. Rogers W. H. Whitlow S. D. DeMers R. (2016). Human performance risks and benefits of adaptive systems on the flight deck. Intl. J. Aviat. Psychol. 26, 15–35. 10.1080/10508414.2016.1226834

[B10] Dorneich M. C. Whitlow S. D. Mathan S. Ververs P. M. Erdogmus D. Pavel M. (2007). Supporting real-time cognitive state classification on a mobile participant. J. Cogn. Eng. Decision Making 1, 240–270. 10.1518/155534307X255618

[B11] Engle M. (2000). Culture in the cockpit—CRM in a multicultural world. J. Air Transport. World Wide 5, 107–114.

[B12] Entin E. B. Entin E. E. (2000). Assessing team situation awareness in simulated military missions, in Proceeding of the Human Factors and Ergonomics Society 44th Annual Meeting (San Diego, CA: Human Factors and Ergonomics Society Press), 73–77.

[B13] Feigh K. M. Dorneich M. C. Hayes C. C. (2012). Toward a characterization of adaptive systems: a framework for researchers and system designers. Hum. Factors 54, 1008–1024. 10.1177/0018720812443983 23397810

[B14] Grech M. R. Neal A. Yeo G. Humphreys M. Smith S. (2009). An examination of the relationship between workload and fatigue within and across consecutive days of work: is the relationship static or dynamic? J. Occup. Health Psychol. 14, 231. 10.1037/a0014952 19586219

[B15] Hancock P. A. Jagacinski R. J. Parasuraman R. Wickens C. D. Wilson G. F. Kaber D. B. (2013). Human-automation interaction research: past, present, and future. Ergon. Design 21, 9–14. 10.1177/1064804613477099

[B16] Hancock P. A. Verwey W. B. (1997). Fatigue, workload and adaptive driver systems. Accident Anal. Prevent. 29, 495–506. 10.1016/S0001-4575(97)00029-8 9248508

[B17] Hart S. G. Wickens C. D. (1990). Workload assessment and prediction, in Manprint, ed Booher H. R. (New York, NY: Springer), 257–296.

[B18] Helmreich R. L. Merritt A. C. Wilhelm J. A. (1999). The evolution of crew resource management training in commercial aviation. Intl. J. Aviat. Psychol. 9, 19–32. 10.1207/s15327108ijap0901_2 11541445

[B19] Helmreich R. L. Merritt A. R. (2001). Culture at Work in Aviation and Medicine: National Organizational and Professional Influences. New York, NY: Routledge.

[B20] Heslegrave R. J. Furedy J. J. (1979). Sensitivities of HR and T-wave amplitude for detecting cognitive and anticipatory stress. Physiol. Behav. 22, 17–23. 10.1016/0031-9384(79)90397-4 451029

[B21] Hockey G. R. J. Briner R. B. Tattersall A. J. Wiethoff M. (1989). Assessing the impact of computer workload on operator stress: the role of system controllability. Ergonomics 32, 1401–1418. 10.1080/00140138908966914 2698802

[B22] Hollan J. Hutchins E. Kirsch D. (2000). Distributed cognition: Toward a new foundation for human-computer interaction research. ACM Transact. Comp. Hum. Interact. 7, 174–196. 10.1145/353485.353487

[B23] Hutchins S. G. Hocevar S. P. Kemple W. G. (1999). Analysis of Team Communications in “Human-in-The-Loop” Experiments in Joint Command and Control. Naval Postgraduate School Monterey Graduate School of Operational and Information Sciences.

[B24] IATA (2016). IATA Forecasts Passenger Demand to Double Over 20 Years. International Air Transport Association (IATA) Press Release 59. Available online at: http://www.iata.org/pressroom/pr/Pages/2016-10-18-02.aspx

[B25] Izzetoglu K. Bunce S. (2004). Functional optical brain imaging using near-infrared during cognitive tasks. Intl. J. Hum. Comp. Interact. 17, 211–227. 10.1207/s15327590ijhc1702_6

[B26] Kalsbeek J. W. H. Ettema J. H. (1963). Scored irregularity of the heart pattern and measurement of perceptual or mental load. Ergonomics 6, 306–307.

[B27] Kanki B. G. (2010). Communication and crew resource management, in Crew Resource Management, eds Kanki B. Helmriech R. Anca J. (San Diego, CA: Academic Press, Inc.), 111–145.

[B28] Kanki B. G. Palmer M. T. Veinott E. (1991). Communication variations related to leader personality, in Proceedings of the Sixth International Symposium on Aviation Psychology (Columbus, OH: Ohio State University), 253–259.

[B29] MacMillan J. Entin E. E. Serfaty D. (2004). Communication Overhead: The Hidden Cost of Team Cognition. Team Cognition: Process and Performance at the Inter- and Intra-Individual Level. Washington, DC: American Psychological Association.

[B30] Malakis S. Kontogiannis T. (2008). Cognitive strategies in emergency and abnormal situations training: implications for resilience in air traffic control, in Proceedings of the 3rd Symposium on Resilience Engineering (Juan-les-Pins).

[B31] Meitinger C. Schulte A. (2009). Human-UAV co-operation based on artificial cognition, in International Conference on Engineering Psychology and Cognitive Ergonomics (Berlin; Heidelberg: Springer), 91–100.

[B32] NextGen (2007). Concept of Operations for the Next Generation Air Transportation System. JOINT Planning and Development Office. Available online at: http://jpdo.gov/library/NextGen_v2.0.pdf

[B33] Page E. B. (1963). Ordered hypotheses for multiple treatments: a significance test for linear ranks. J. Am. Stat. Assoc. 58, 216–230. 10.2307/2282965

[B34] Porter C. O. Webb J. W. Gogus C. I. (2010). When goal orientations collide: effects of learning and performance orientation on team adaptability in response to workload imbalance. J. Appl. Psychol. 95, 935. 10.1037/a0019637 20718514

[B35] Prinzell L. J. Freeman F. G. Scerbo M. W. Mikulka P. J. Pope A. T. (2003). Effects of a psychophysiological system for adaptive automation on performance, workload, and the event-related potential p300 component. Hum. Factors 45, 601–614. 10.1518/hfes.45.4.601.27092 15055457

[B36] Quon L. (2010). Airspace Systems Program: NextGen Systems Analysis, Integration and Evaluation Project Plan. NASA Report ID 20110011155.

[B37] Ruffell Smith H. P. (1979). A Simulator Study of the Interaction of Pilot Workload with Errors, Vigilance, and Decisions. NASA Ames Research Center, Moffett Field, CA. NASA Technical Memorandum 78482.

[B38] Salas E. Cooke N. J. Rosen M. A. (2008). On teams, teamwork, and team performance: discoveries and developments. Hum. Fact. 50, 540–547. 10.1518/001872008X288457 18689065

[B39] Sarter N. B. Woods D. D. Billings C. E. (1997). Automation Surprises, in Handbook of Human Factors and Ergonomics, 2nd Edn. ed Salvendy G. (New York, NY: Wiley), 1926–1943.

[B40] Scerbo M. W. (1996). Theoretical perspectives on adaptive automation, in Automation and Human Performance: Theory and Applications, eds Parasuraman R. Mouloua M. (Mahwah, NJ: Lawrence Erlbaum Associates), 37–63.

[B41] SESAR Consortium (2006). D1 Air Transport Framework: The current situation. SESAR Definition Phase – Milestone Deliverable 1. Available online at: http://www.sesarju.eu/gallery/content/public/DLM-0602-001-03-00.pdf

[B42] Smith-Jentsch K. A. Johnston J. H. Payne S. C. (1998). Measuring team-related expertise in complex environments, in Making Decisions Under Stress: Implications for Individual and Team Training, eds Cannon-Bowers J. A. Salas E. (Washington, DC: American Psychological Association), 61–87. 10.1037/10278-003

[B43] Wickens C. D. (1986). Gain and energetics in information processing theory, *in* Energetics and Human Information Processing, eds Hockey G. R. J. Gaillard A. W. K. Coles M. G. H. (Dordrecht: Martinus Nijhoff), 373–389.

[B44] Wildervanck C. Mulder G. Michon J. A. (1978). Mapping mental load in car driving. Ergonomics 21, 225–229. 10.1080/00140137808931717 668666

[B45] Williams H. P. Tham M. Wickens C. D. (1993). Workload management and geographic disorientation in aviation incidents: a review of the ASRS data base, in Proceedings of the Seventh International Symposium on Aviation Psychology, ed Jensen R. S. (Columbus, OH: Ohio State University, Department of Aviation), 960–964.

[B46] Wilson G. F. Eggemeier F. T. (1991). Physiological measures of workload in multi-task environments, in Multiple-task Performance, ed Damos D. (London: Taylor & Francis), 329–360

[B47] Wilson G. Russell C. (2003). Operator functional state classification using multiple psychophysiological features in an air traffic control task. Hum. Factors 45, 381–389. 10.1518/hfes.45.3.381.27252 14702990

